# Diiodine-Induced Dimensionality Evolution in Two Antimony(III) Halides for Optimal-Bandgap Photovoltaics

**DOI:** 10.3390/ma19143038

**Published:** 2026-07-14

**Authors:** Xiaoting Liu, Jingjing Liu, Caiting Ji, Yanan Qiao, Chunqing Hou, Xiaoxu Bo

**Affiliations:** 1 School of Energy Engineering, Shanxi College of Technology, Shuozhou 036000, China; liuxiaoting@sxct.edu.cn (X.L.); liujingjing@sxct.edu.cn (J.L.); jicaiting@sxct.edu.cn (C.J.); qiaoyanan@sxct.edu.cn (Y.Q.); 2 Sinopec Beihua Institute (Tianjin) Technology Development Co., Ltd., Tianjin 300452, China; houchq.bjhy@sinopec.com; 3 State Key Laboratory of Supramolecular Structure and Materials, College of Chemistry, Jilin University, Changchun 130012, China; 4 Department of Agriculture and Biotechnology, Wenzhou Vocational College of Science and Technology, Wenzhou 325006, China

**Keywords:** antimony-based hybrid, crystal structure, theoretical study, electronic structure, I_2_ molecules

## Abstract

**Highlights:**

- Diiodine-induced host-guest assembly drives a 1D-to-0D evolution in antimony(III) halides.
- This structural transformation narrows the optical bandgap to an optimal 1.46 eV, approaching the Shockley-Queisser limit.
- Strong sp-orbital hybridization ensures robust visible-light harvesting, with absorption coefficients exceeding 10^4^ cm^−1^.
- The resulting 0D complex exhibits high intrinsic thermal stability and remains structurally intact after a 6-month ambient exposure.

**Abstract:**

Developing lead-free organic-inorganic hybrid metal halides with strong light-harvesting capabilities and superior stability, while precisely tuning their crystalline phases and electronic structures, remains a key challenge in optoelectronics. Herein, we report a neutral iodine-induced structural transition from a 1D chain-like (C_6_H_11_NH_3_)_2_SbI_5_ architecture to a 0D dimeric (C_6_H_11_NH_3_)_3_[Sb_2_I_9_]·I_2_ supramolecular host-guest complex. This transformation is achieved via a controlled solution-cooling crystallization process, yielding high-quality bulk single crystals. Crystallographic analysis reveals that N–H···I hydrogen-bonding networks stabilize the organic cations, while halogen bonding interactions anchor the I_2_ guests within the lattice cavities of the [Sb_2_I_9_]^3−^ dimeric host. Experimental characterizations, including XRD, TGA, and XPS, confirm the high phase purity and thermal stability of the (C_6_H_11_NH_3_)_3_[Sb_2_I_9_]·I_2_ hybrid and determine its electronic band structure. To further elucidate the underlying mechanisms, theoretical calculations were performed, revealing that strong sp-orbital hybridization yields a high absorption coefficient. The associated dimensional transition narrows the direct optical bandgap to 1.46 eV, approaching the Shockley-Queisser limit and demonstrating strong potential for visible-light harvesting. This work elucidates the role of supramolecular host-guest interactions in modulating the lattice evolution of lead-free antimony-based materials, presenting halogen guest engineering as an effective approach for optoelectronic material design.

## 1. Introduction

In recent years, organic-inorganic hybrid lead halide perovskites have shown great promise in the fields of solar cells, light-emitting diodes, nonlinear photonics, X-ray scintillators, circularly polarized light detectors, and anti-counterfeiting, benefiting from their structural diversity, tunable exciton binding energies, and superior optoelectronic properties, as well as their facile solution processability and low-cost synthesis [1,2,3,4,5,6,7,8]. However, their large-scale commercialization is hindered by the intrinsic toxicity of lead and poor long-term stability under humid and thermal conditions [2,3,4,9]. Therefore, developing structurally stable and lead-free alternatives has become an urgent priority.

Given that the Group VA cations Bi^3+^ and Sb^3+^ possess stable +3 oxidation states and the same active ns^2^ lone-pair state as Pb^2+^, Sb-based and Bi-based perovskites are emerging as promising candidates for next-generation photovoltaic applications [10,11,12,13,14]. The stereochemically active lone-pair electrons of Sb^3+^ endow the inorganic framework with an inherent structural flexibility, enabling A-site steric or geometric modulation to effectively direct the structural transformation of fundamental polyhedral building blocks, such as the seesaw-like [SbI_4_]^−^, square pyramidal [SbI_5_]^2−^, and octahedral [SbI_6_]^3−^ geometries [15,16,17]. Furthermore, because the +3 oxidation states of Bi and Sb differ from the +2 state of Pb, the stoichiometry and spatial arrangement of these halides are dictated accordingly, thereby facilitating their crystallization into low-dimensional phases, typically A_3_M_2_X_9_ [18,19,20].

Consequently, the polyhedral rearrangement induces the dimensional evolution that significantly alters the degree of orbital overlap within the lattice, thereby enabling the effective modulation of exciton binding energies, optical bandgaps, and carrier dynamics. While materials such as Cs_3_Sb_2_I_9_ and Rb_3_Sb_2_I_9_ exhibit pronounced variations in optoelectronic properties across different low-dimensional phases, conventional antimony-based halides are still limited by wide bandgaps and restricted charge transport [21,22,23]. Therefore, the exploration of novel structure-directing strategies to induce crystal structure evolution for the systematic optimization of optoelectronic performance is highly desirable.

Sb-based perovskite-inspired halides possess strong spin–orbit coupling and high polarizability. However, their power conversion efficiency (PCE) remains limited to ~4% [24,25]. This limitation stems primarily from poor charge transport and a wide bandgap (>2 eV), the latter of which severely restricts visible-light absorption. To effectively enhance the optical absorption of these materials, their bandgaps must be narrowed to ~1.5 eV to ideally match the solar spectrum [26]. Consequently, the exploration of novel structure-directing factors to induce lattice evolution has emerged as a key strategy, enabling precise bandgap engineering to address the optoelectronic limitations inherent in lead-free halides.

The fundamental building units of metal halides facilitate the construction of a wide variety of iodoantimonate compounds via self-condensation and self-assembly [27]. To address the inherent performance limitations of these materials, molecular-scale lattice engineering and compositional modulation strategies have been extensively adopted [28,29,30]. Recent studies have demonstrated that incorporating neutral iodine (I_2_) molecules or constructing polyiodide networks within the halide lattice can modulate the electronic band structure, thereby narrowing the optical bandgap, broadening the spectral absorption range, and significantly enhancing the intrinsic electrical conductivity through strengthened intra-lattice electronic delocalization [31,32,33,34,35]. While I_2_ incorporation in bismuth and antimony halides has been reported, studies on I_2_-induced dimensional or structural evolution in hybrid antimony halides templated by bulky organic cations remain limited, and the underlying optoelectronic mechanisms are still unclear.

Herein, by employing bulky cyclohexylammonium as organic template cations and introducing neutral iodine molecules as guests, we synthesized two novel hybrid single crystals, namely the one-dimensional (1D) chain structure, (C_6_H_11_NH_3_)_2_SbI_5_, and the zero-dimensional (0D) host-guest assembly, (C_6_H_11_NH_3_)_3_[Sb_2_I_9_]·I_2_. Subsequently, we systematically investigated the structural transition from the 1D chain to the 0D dimer, thereby clarifying the dimensionality reduction mechanism at the molecular level. Specifically, single-crystal X-ray diffraction (SCXRD) analysis of (C_6_H_11_NH_3_)_3_[Sb_2_I_9_]·I_2_ confirmed a synergistic interaction between the hydrogen-bonding network and halogen bonds, effectively anchoring the host framework and stabilizing the guest molecules. Furthermore, thermal analyses and spectroscopic measurements, including UV-Vis-NIR and XPS, demonstrated that this dimensional tuning effectively regulates the thermal stability and optical bandgaps of the halides. Additionally, density functional theory (DFT) calculations elucidated the band structure evolution and electron localization effects induced by this structural variance, revealing the underlying physical mechanisms governing optoelectronic property modulation at the electronic level. In summary, this study enriches the structural diversity of low-dimensional antimony-based hybrid halides and establishes guest molecule engineering as an effective strategy for tailoring the optical properties of lead-free optoelectronic semiconductors.

## 2. Materials and Methods

### 2.1. Materials

Antimony (III) iodide (98%), cyclohexylammonium iodide (99%), and hydriodic acid (57 wt.% in H_2_O) were obtained from Aladdin (Shanghai, China) and used without further purification.

### 2.2. Growth of (C_6_H_11_NH_3_)_2_SbI_5_ and (C_6_H_11_NH_3_)_3_[Sb_2_I_9_]·I_2_ Bulk Single Crystals

For the preparation of (C_6_H_11_NH_3_)_2_SbI_5_, SbI_3_ (0.50 mmol, 0.251 g) and C_6_H_13_N•HI (1.00 mmol, 0.227 g) were dissolved in 1 mL of aqueous HI (57 wt.%). The solution was maintained at 383 K under continuous stirring for 1 h. Controlled cooling to 298 K at a constant rate of 1 °C/h yielded the target bulk crystals. Regarding (C_6_H_11_NH_3_)_3_[Sb_2_I_9_]·I_2_, two synthetic routes were investigated. Initially, I_2_ (0.25 mmol, 0.064 g) was added directly to the precursor solution prepared for the first compound. Although this method produced the desired phase, the isolated yield was relatively low. To improve the product yield, a stoichiometric approach based on a 2:3 ratio was examined. In this procedure, SbI_3_ (0.50 mmol, 0.251 g), C_6_H_13_N•HI (0.75 mmol, 0.170 g), and I_2_ (0.25 mmol, 0.064 g) were dissolved in aqueous HI (1 mL, 57 wt.%). Under identical thermal conditions (383 K for 1 h, followed by cooling at 1 °C/h to 298 K), (C_6_H_11_NH_3_)_3_[Sb_2_I_9_]·I_2_ crystals were obtained. The resulting crystals were separated from the mother liquor, washed thoroughly with diethyl ether, and dried under vacuum.

### 2.3. Characterization

The absorption spectra were recorded using a UV-VIS-NIR spectrophotometer (UV-3600, Shimadzu, Kyoto, Japan). Room-temperature (293 K) SCXRD measurements were conducted on a Rigaku R-AXIS RAPID diffractometer (Rigaku, Tokyo, Japan; Mo Kα radiation, graphite monochromator, λ = 0.71073 Å). Data were empirically corrected for absorption. The structure was solved by direct methods and refined by full-matrix least-squares on F^2^ (SHELXTL-2014 program [36]). Anisotropic refinement was performed for all non-hydrogen atoms. Additional crystallographic details are available from the Cambridge Crystallographic Data Center (CCDC) under deposition numbers 2563200 and 2563201. Powder X-ray diffraction (PXRD) data were collected on a PANalytical X’Pert Pro diffractometer (PANalytical B.V., Almelo, The Netherlands) equipped with a Cu Kα source (λ = 1.54059 Å, 40 kV, 40 mA) at room temperature (293 K). The 2θ scanning range was set to 5–50° (step size: 0.02°). Thermal stability was evaluated on a TA Instruments SDT Q500 analyzer (New Castle, DE, USA). Data were collected from 40 °C to 800 °C (heating rate: 10 °C/min) in a dynamic N_2_ atmosphere (100 mL/min). X-ray photoelectron spectroscopy (XPS) measurements were conducted on a VG Scienta R3000 system (VG Scienta, Uppsala, Sweden) to determine surface chemical compositions. The instrument operated at a base pressure of 1 × 10^−10^ mbar with a monochromatic Al Kα X-ray source (1486.6 eV).

### 2.4. Theoretical Calculation

All theoretical calculations, including structural optimizations and electronic property evaluations, were performed within the plane-wave pseudopotential DFT framework using the CASTEP-2020 package [37]. The exchange-correlation interactions were described by the generalized gradient approximation (GGA) employing the Perdew–Burke–Ernzerhof (PBE) functional [38]. Valence configurations of Sb (5s^2^5p^3^), I (5s^2^5p^5^), N (2s^2^2p^3^), C (2s^2^2p^2^), and H (1s^1^) were applied. Additionally, spin–orbit coupling (SOC) and van der Waals (vdW) corrections were employed to capture the properties of heavy elements and host-guest interactions. Geometrical optimizations relied on the fixed experimental X-ray lattice parameters while fully relaxing the internal atomic coordinates, using an 820 eV cutoff energy and a 10^−5^ eV/atom convergence threshold. The relaxed structures were subsequently used to derive the band structures and the density of states (self-consistent convergence: 10^−6^ eV/atom). The optical properties were theoretically analyzed by computing the frequency-dependent dielectric function, $\varepsilon \left(\omega \right)=\varepsilon_{1}\left(\omega \right)+i\varepsilon_{2}\left(\omega \right)$. Based on its real ($\varepsilon_{1}\left(\omega \right)$) and imaginary ($\varepsilon_{2}\left(\omega \right)$) parts, the absorption coefficient ($\alpha \left(\omega \right)$) was evaluated as $\alpha \left(\omega \right)=\left(\omega /c\right){\left[2{\left(\varepsilon_{1}^{2}\left(\omega \right)+\varepsilon_{2}^{2}\left(\omega \right)\right)}^{1/2}-2\varepsilon_{1}\left(\omega \right)\right]}^{1/2}$, where *c* is the speed of light [21].

## 3. Results and Discussion

### 3.1. Crystal Structures of (C_6_H_11_NH_3_)_2_SbI_5_ and (C_6_H_11_NH_3_)_3_[Sb_2_I_9_]·I_2_ Hybrids

**Figure 1:**
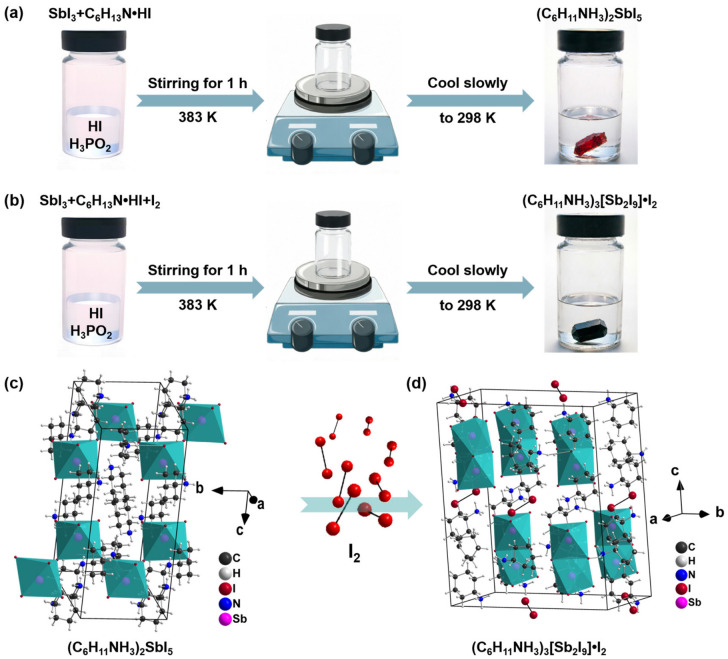
(**a**,**b**) Schematic diagrams of the preparation for (C_6_H_11_NH_3_)_2_SbI_5_ and (C_6_H_11_NH_3_)_3_[Sb_2_I_9_]·I_2_ crystals. (**c**,**d**) Crystal structures illustrating the l_2_-induced transformation from (C_6_H_11_NH_3_)_2_SbI_5_ and (C_6_H_11_NH_3_)_3_[Sb_2_I_9_]·I_2_. Black, gray, red, blue, and magenta spheres represent C, H, I, N, and Sb atoms, respectively.

As illustrated in Figure 1a,b, the two Sb-based halide hybrids, (C_6_H_11_NH_3_)_2_SbI_5_ and (C_6_H_11_NH_3_)_3_[Sb_2_I_9_]·I_2_, were successfully synthesized via a controlled solution-cooling crystallization method. Their precise crystal structures were subsequently determined using single-crystal X-ray diffraction. Specifically, (C_6_H_11_NH_3_)_2_SbI_5_ crystallizes in the monoclinic space group *P*2_1_/*n* (No. 14) at room temperature, with lattice parameters of *a* = 13.85(3) Å, *b* = 8.72(2) Å, *c* = 20.59(4) Å, and β = 95.2580(10)°. Detailed crystallographic data are summarized in Table S1. As illustrated in Figure 1c, each Sb center is coordinated by I^−^ ions to form an SbI_6_ octahedron, which connects with adjacent octahedra via shared corners to construct infinite zigzag chains extending along the b axis. Within this arrangement, large intercalated cyclohexylammonium cations sterically isolate these individual anionic chains, establishing a characteristic 1D structure at the molecular level.

Driven by the stereochemically active lone pairs of the Sb^3+^ ions, these inorganic polyhedra exhibit pronounced structural distortions, resulting in asymmetric bond lengths and bond angles that deviate from ideal symmetry. The polyhedral distortion plays a fundamental role in modulating carrier transport and exciton-phonon interactions. Specifically, within (C_6_H_11_NH_3_)_2_SbI_5_, the SbI_6_ octahedra adopt a highly distorted geometry featuring Sb–I bond lengths of 2.81–3.07 Å and I–Sb–I bond angles of 90.58–171.22° (Table S2). While the angles deviate from the ideal 90° and 180°, the geometric parameters remain comparable to those observed in other 1D networks featuring corner-sharing [SbI_5_]^2−^ structures [15].

Large cyclohexylammonium cations (C_6_H_11_NH_3_^+^) occupy the interstitial voids between adjacent inorganic chains, where an extensive network of N–H···I hydrogen bonds anchors these organic cations to the iodide framework with a minimum interaction distance of 2.73 Å. These strong supramolecular interactions significantly enhance the structural stability of the crystalline lattice by linking the individual one-dimensional (1D) chains into a robust three-dimensional (3D) supramolecular network. Despite this extended 3D connectivity, the 1D inorganic motifs retain inherent quantum confinement, which localizes excitons within the inorganic framework, thereby endowing the material with distinctive optoelectronic properties.

In contrast, (C_6_H_11_NH_3_)_3_[Sb_2_I_9_]·I_2_ crystallizes in the monoclinic space group *C*2/*c*, with lattice parameters of *a* = 8.54(2) Å, *b* = 21.98(6) Å, *c* = 23.32(7) Å, and β = 90.193(1)° (Table S1). As illustrated in Figure 1d, each Sb center is octahedrally coordinated by six I^−^ ions, forming an [SbI_6_]^3−^ unit that connects with an adjacent octahedron via face-sharing to assemble the typical [Sb_2_I_9_]^3−^ dimeric anion, which is sterically separated from neighboring dimers by cyclohexylammonium cations. The asymmetric unit of (C_6_H_11_NH_3_)_3_[Sb_2_I_9_]·I_2_ comprises one discrete [Sb_2_I_9_]^3−^ inorganic anion, three C_6_H_11_NH_3_^+^ cations, and one guest I_2_ molecule, which occupies the interstitial voids between the dimeric anions to form a characteristic 0D architecture, thereby establishing a host-guest supramolecular system stabilized by extensive intermolecular interactions (Figure 1d).

**Figure 2:**
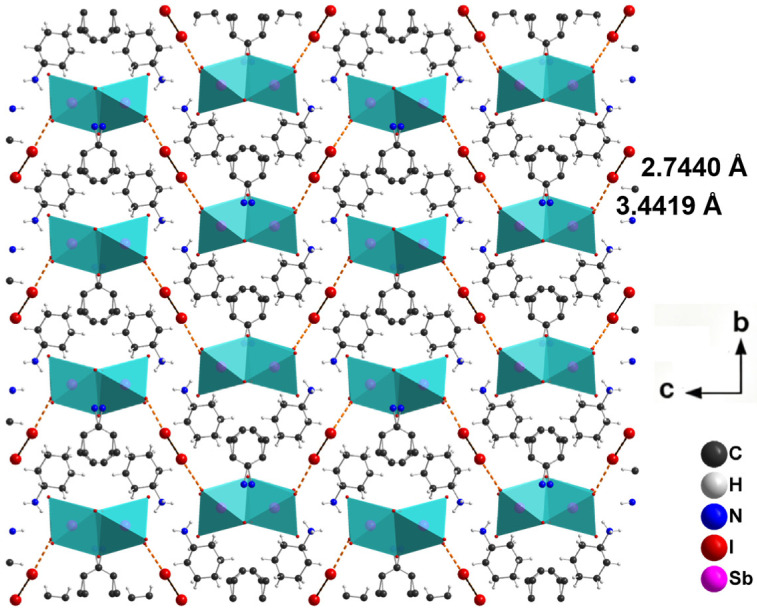
Crystal structures of (C_6_H_11_NH_3_)_3_[Sb_2_I_9_]·I_2_ along the c-axis; orange dashed lines denote I···I halogen bonding.

Within the [Sb_2_I_9_]^3−^ dimer, the SbI_6_ octahedra exhibit a highly distorted geometry with Sb–I bond lengths of 2.81–3.19 Å and I–Sb–I bond angles of 84.93–178.51° (Table S3), representing deviations from ideal symmetry that remain consistent with those observed in other face-sharing dimeric architectures [19,20,21]. In the crystal lattice of (C_6_H_11_NH_3_)_3_[Sb_2_I_9_]·I_2_, the guest iodine molecule exhibits an intramolecular I–I bond length of 2.74 Å, which is slightly elongated compared to the 2.72 Å length of free molecular iodine (Figure 2). Structurally, these guest molecules bridge the adjacent [Sb_2_I_9_]^3−^ dimers via rigid I···I halogen bonds with an intermolecular distance of 3.44 Å, which is markedly longer than an intramolecular I–I covalent bond but remains considerably shorter than the sum of the corresponding van der Waals radii. Furthermore, the nearly linear I–I···I angle of 175.21° confirms the highly directional nature characteristic of halogen bonding.

Consequently, the supramolecular framework of (C_6_H_11_NH_3_)_3_[Sb_2_I_9_] acts as a robust host matrix, within which the guest I_2_ molecules are securely stabilized through extensive noncovalent interactions, consistent with analogous host-guest systems. Specifically, the host-guest assembly is reinforced by both I···I halogen bonds and N–H···I hydrogen bonds, the latter arising from interactions between the amino groups of the C_6_H_11_NH_3_^+^ cations and the iodine atoms of the SbI_6_ octahedra. Notably, the I···I halogen bonding is energetically comparable to strong hydrogen bonds, highlighting the critical role of halogen bonding interactions in the self-assembly process [17]. Together, these strong noncovalent interactions significantly enhance structural stability by effectively linking the discrete 0D units into a robust 3D supramolecular network.

### 3.2. Optical Properties of (C_6_H_11_NH_3_)_2_SbI_5_ and (C_6_H_11_NH_3_)_3_[Sb_2_I_9_]·I_2_ Hybrids

**Figure 3:**
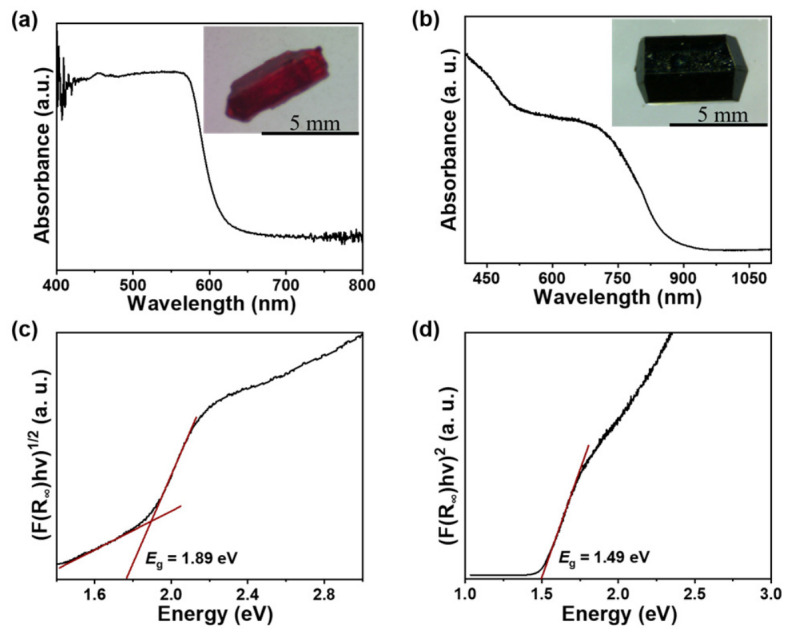
(**a**,**b**) UV-vis absorption spectra of (C_6_H_11_NH_3_)_2_SbI_5_ and (C_6_H_11_NH_3_)_3_[Sb_2_I_9_]·I_2_, respectively. The insets show optical photographs of the corresponding single crystals. (**c**,**d**) Corresponding Tauc plots for the bandgap (*E_g_*) of the two Sb-based hybrids.

The light absorption properties of the two synthesized hybrids were investigated by measuring their UV-vis diffuse reflectance spectra. These reflectance data were then converted to absorbance using the Kubelka-Munk equation. Guided by the electronic band structure calculations, optical bandgaps were extracted from the corresponding Tauc plots. Specifically, the 1D (C_6_H_11_NH_3_)_2_SbI_5_ compound displays an indirect bandgap of 1.89 eV, which corresponds to an absorption onset at approximately 656 nm (Figure 3a,c). In contrast, the I_2_-incorporated 0D (C_6_H_11_NH_3_)_3_[Sb_2_I_9_]·I_2_ structure features a significant redshift to ~838 nm, which results in a reduced direct bandgap of 1.49 eV (Figure 3b,d). Comprehensive details on the calculation procedure and a comparison of the direct and indirect Tauc plots are provided in the Supporting Information (Figure S2). Distinct from typical Sb-based hybrid halides with wide bandgaps exceeding 1.8 eV, this I_2_-containing compound exhibits a bandgap very close to that of the benchmark MAPbI_3_ (~1.5 eV). The macroscopic appearances of the crystals match well with their absorption edges, shifting from dark red in the I_2_-free crystal to black in the I_2_-incorporated compound, yielding a broad absorption that nearly covers the entire visible light spectrum.

The significant bandgap reduction primarily stems from the structural evolution of the Sb-I coordination units. Specifically, the inorganic framework in (C_6_H_11_NH_3_)_2_SbI_5_ exhibits a 1D chain composed of corner-sharing polyhedra, while the I_2_-incorporated (C_6_H_11_NH_3_)_3_[Sb_2_I_9_]·I_2_ hybrid features a 0D dimer architecture formed through face-sharing. To elucidate the fundamental origin of this optically observed variation, first-principles calculations were performed.

### 3.3. Electronic Band Structures of (C_6_H_11_NH_3_)_2_SbI_5_ and (C_6_H_11_NH_3_)_3_[Sb_2_I_9_]·I_2_ Hybrids

**Figure 4:**
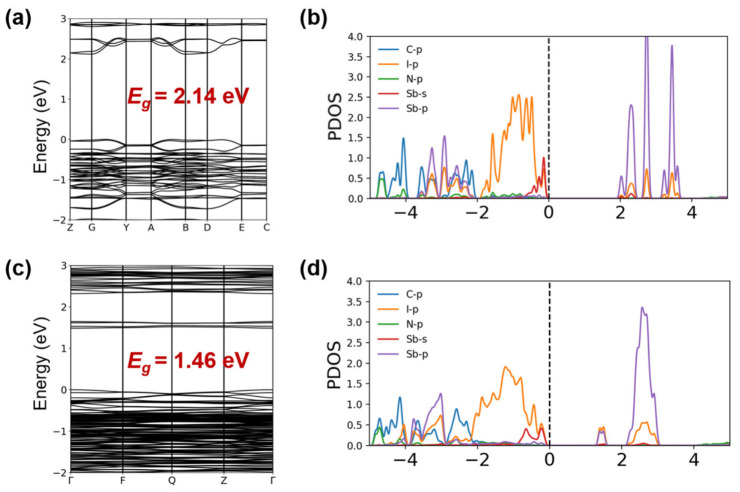
(**a**,**c**) Calculated band structures of (C_6_H_11_NH_3_)_2_SbI_5_ and (C_6_H_11_NH_3_)_3_[Sb_2_I_9_]·I_2_ hybrids, respectively. (**b**,**d**) Corresponding partial density of states (PDOS) plots.

The calculations reveal that both structures exhibit nearly direct bandgap characteristics, with calculated bandgaps of 2.14 eV for the 1D chain and 1.46 eV for the 0D dimer (Figure 4a,c), consistent with the experimental narrowing trend. Projected density of states (PDOS) analysis demonstrates that for both structures, the valence band maximum (VBM) originates from antibonding states induced by hybridization between I 5p and Sb 5s orbitals, whereas the conduction band minimum (CBM) primarily derives from Sb 5p orbitals (Figure 4b,d). To further elucidate the electronic evolution accompanying this structural transformation, the density of states was evaluated. In the 1D structure, the energy bands exhibit dispersion reflecting electronic delocalization along the extended inorganic chains, whereas the band structure of the 0D dimer exhibits flat energy bands, indicating quantum confinement and electronic localization within the isolated [Sb_2_I_9_]^3−^ dimer. The localized states at the band edges shift toward the Fermi level due to enhanced intra-dimer orbital coupling and quantum-mechanical splitting within the face-sharing dimeric unit, which supports the microscopic orbital hybridization mechanism and confirms the origin of the bandgap reduction.

### 3.4. XPS Analysis of (C_6_H_11_NH_3_)_2_SbI_5_ and (C_6_H_11_NH_3_)_3_[Sb_2_I_9_]·I_2_ Hybrids

**Figure 5:**
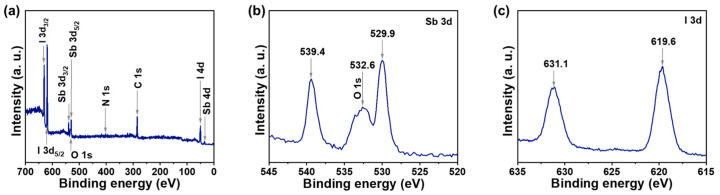
XPS spectra (Al Kα) of the as-prepared (C_6_H_11_NH_3_)_3_[Sb_2_I_9_]·I_2_ crystals. (**a**) Full survey spectrum. (**b**) High-resolution XPS spectra of the Sb 3d and (**c**) I 3d regions.

The electronic structure is fundamentally supported by the valence stability of the inorganic components. To investigate the surface chemical composition and elemental valence states, X-ray photoelectron spectroscopy (XPS) measurements were performed using Al Kα radiation. The survey spectrum reveals characteristic peaks for C, N, Sb, and I, consistent with the theoretical stoichiometry, accompanied by trace amounts of surface-adsorbed oxygen (Figure 5a). The absence of other detectable impurity peaks indicates the high surface purity of the as-prepared crystals. Furthermore, the high-resolution Sb 3d spectrum exhibits a spin–orbit doublet at 529.9 eV (Sb 3d_5/2_) and 539.4 eV (Sb 3d_3/2_), confirming the Sb^3+^ oxidation state (Figure 5b). Meanwhile, the high-resolution I 3d spectrum displays a characteristic doublet at 619.6 eV (I 3d_5/2_) and 631.1 eV (I 3d_3/2_), corresponding to I^−^ ions (Figure 5c). While a weak signal at 532.6 eV is attributed to trace amounts of surface-adsorbed oxygen, the absence of detectable oxidation products, such as Sb^5+^ or high-valence iodine species, rules out significant surface oxidation in the as-prepared state. This observed valence stability of Sb^3+^ and I^−^ ensures local charge neutrality and lattice integrity, which provide a solid chemical foundation for its potential applications. Raman spectroscopy was further performed, displaying a spectrum that exhibits a sharp peak at 177 cm^−1^ (Figure S3). This spectral feature, which presents a minor redshift of 3 cm^−1^ from the standard I–I stretching vibration of solid I_2_ (~180 cm^−1^), confirms that the neutral I_2_ molecule is incorporated into the lattice and engages in secondary halogen bonding with the host. Furthermore, the weaker bands below 150 cm^−1^ correspond to the vibrational modes of the [Sb_2_I_9_]^3─^ dimer, which is consistent with the host-guest structural model.

Given that the VBM originates from the hybridized Sb^3+^ 5s and I^−^ 5p orbitals, oxidation to Sb^5+^ would deplete the 5s lone-pair electrons, which inherently lowers the VBM and broadens the bandgap. The narrow 1.46 eV bandgap matches the solar spectrum for visible-light harvesting, whereas the wider bandgap counterpart is suited for short-wavelength photodetection. Furthermore, while the organic cations make no direct contribution to the frontier bands, their steric hindrance modulates the inorganic coordination geometry, providing a basis for performance optimization via cation engineering.

### 3.5. Stability of (C_6_H_11_NH_3_)_2_SbI_5_ and (C_6_H_11_NH_3_)_3_[Sb_2_I_9_]·I_2_ Hybrids

**Figure 6:**
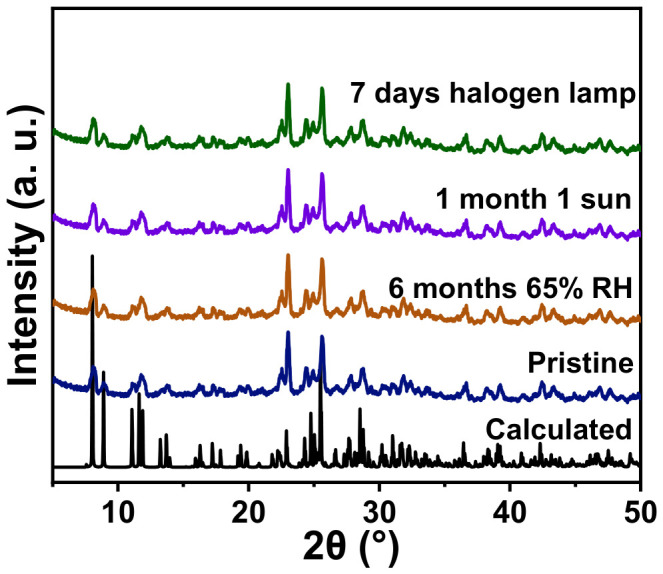
PXRD patterns of (C_6_H_11_NH_3_)_3_[Sb_2_I_9_]·I_2_ after exposure to various environmental conditions.

As long-term stability is essential for practical photovoltaic applications, the structural stability of (C_6_H_11_NH_3_)_3_[Sb_2_I_9_]·I_2_ was systematically evaluated under thermal, moisture, and light conditions. Thermogravimetric analysis (TGA) reveals a decomposition onset temperature of 152 °C, indicating high intrinsic thermal stability (Figure S1). Notably, the unencapsulated material exhibits high environmental and photostability. The powder X-ray diffraction (PXRD) patterns remain identical to those of the pristine sample and are consistent with the calculated phase even after a 6-month exposure to ambient air (65% RH), 1 month of continuous 1-sun illumination, and 7 days of intense halogen light irradiation, which demonstrates the absence of any structural degradation or phase transition (Figure 6).

Fundamentally, the enhanced stability stems from the dense host-guest supramolecular network and the highly hydrophobic alicyclic rings of the cyclohexylammonium cations, which synergistically shield the inorganic framework against environmental stressors.

### 3.6. Optoelectronic and Photovoltaic Prospects of (C_6_H_11_NH_3_)_2_SbI_5_ and (C_6_H_11_NH_3_)_3_[Sb_2_I_9_]·I_2_ Hybrids

**Figure 7:**
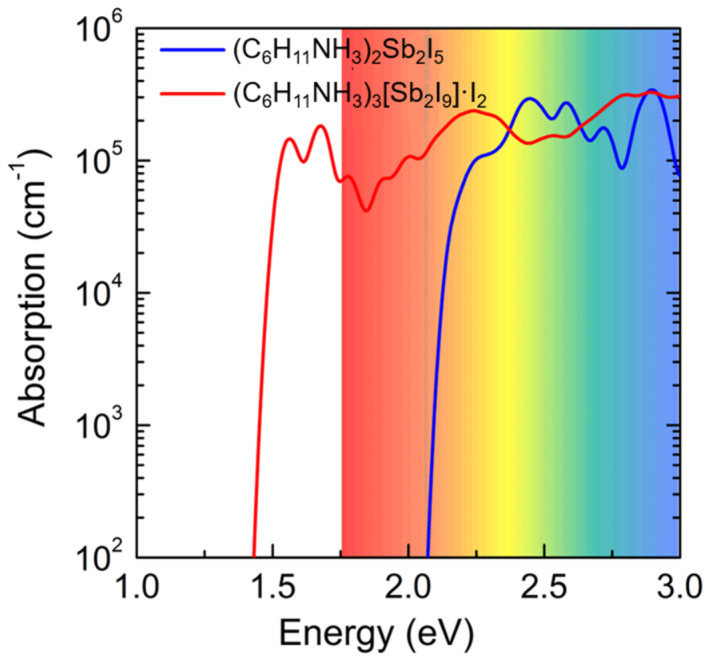
Calculated absorption spectra of the Sb-based halides (C_6_H_11_NH_3_)_2_SbI_5_ and (C_6_H_11_NH_3_)_3_[Sb_2_I_9_]·I_2_.

Based on the calculated electronic structures, the optical absorption coefficients of (C_6_H_11_NH_3_)_2_SbI_5_ and (C_6_H_11_NH_3_)_3_[Sb_2_I_9_]·I_2_ were evaluated (Figure 7). Both compounds exhibit absorption coefficients exceeding 10^4^ cm^−1^ in the visible region, comparable to the prototypical lead halide perovskite CH_3_NH_3_PbI_3_. As revealed by the density-of-states analysis, this light-harvesting capability originates from the strong sp-orbital hybridization governing the fundamental optical transitions. Analogous to CH_3_NH_3_PbI_3_, where optical transitions arise from the (I 5p + Pb 6s) to Pb 6p states, such orbital hybridization yields high transition probabilities responsible for the strong optical absorption.

Furthermore, the structural transformation from the 1D chain to the 0D dimer dictates distinct optoelectronic functionalities. The 0D (C_6_H_11_NH_3_)_3_[Sb_2_I_9_]·I_2_ features a narrow bandgap of 1.46 eV, which closely approaches the theoretical Shockley-Queisser limit (~1.34 eV). The resulting extended absorption in the low-energy region facilitates efficient visible-light harvesting. Although the narrow bandgap provides strong light absorption, future studies on charge mobility and device performance are needed to assess its photovoltaic potential. In contrast, the 1D (C_6_H_11_NH_3_)_2_SbI_5_, with its wider 2.14 eV bandgap, exhibits a correspondingly blue-shifted absorption onset. Despite limiting broadband absorption, this wider bandgap provides a distinct transparency window. Similarly, further empirical charge-transport and device characterizations are essential to validate its potential suitability for specific applications, such as short-wavelength photodetectors.

## 4. Conclusions

In summary, we demonstrate a crystal dimensionality evolution induced by I_2_ guest molecules, wherein the 1D chain-like (C_6_H_11_NH_3_)_2_SbI_5_ lattice transitions into a 0D (C_6_H_11_NH_3_)_3_[Sb_2_I_9_]·I_2_ supramolecular host-guest inclusion complex. Structurally, noncovalent interactions, primarily hydrogen and halogen bonds, drive this lattice reconstruction, confining the I_2_ and C_6_H_11_NH_3_^+^ guests within the host framework. Consequently, this I_2_-induced structural transition confers high stability to the complex while tailoring its optical bandgap and electronic band structure. While the tailored bandgap is highly advantageous for light harvesting, further charge-transport measurements and actual device studies are required to confirm its practical photovoltaic potential. Nevertheless, this work establishes that supramolecular host-guest interactions can systematically modulate the structural phase, providing a rational design strategy forlow-dimensional antimony-based hybrid materials.

## Data Availability

The original contributions presented in this study are included in the article’s Supplementary Material. Further inquiries can be directed to the corresponding author.

## Supplementary Materials

The following supporting information can be downloaded at https://www.mdpi.com/article/10.3390/ma19143038/s1. Table S1: A summary of crystal data and structure refinements of (C_6_H_11_NH_3_)_2_SbI_5_ and (C_6_H_11_NH_3_)_3_[Sb_2_I_9_]·I_2_. Table S2: Selected bond lengths (Å) and bond angles (°) for (C_6_H_11_NH_3_)_2_SbI_5_. Table S3: Selected bond lengths (Å) and bond angles (°) for (C_6_H_11_NH_3_)_3_[Sb_2_I_9_]·I_2_. Figure S1: TGA curve (black) and DSC curve (blue) of (C_6_H_11_NH_3_)_3_[Sb_2_I_9_]·I_2_ powder samples. Figure S2: Tauc plots for the bandgap (*E*_g_) of (a) (C_6_H_11_NH_3_)_2_SbI_5_ and (b) (C_6_H_11_NH_3_)_3_[Sb_2_I_9_]·I_2_. Figure S3: Raman spectrum of (C_6_H_11_NH_3_)_3_[Sb_2_I_9_]·I_2_.
