# Supplementary material for: Diiodine-Induced Dimensionality Evolution in Two Antimony(III) Halides for Optimal-Bandgap Photovoltaics
Source: Materials (Basel). 2026 Jul 14;19(14):3038. doi: 10.3390/ma19143038 (PMC13413734; doi:10.3390/ma19143038)
Supplement: Supplementary file 1 [file materials-19-03038-s001.zip › materials-4391737-supplementary.pdf]

Supplementary Information

Diiodine-Induced Dimensionality Evolution in Two Antimony(III) Halides for Optimal-Bandgap Photovoltaics

Table S1. A summary of crystal data and structure refinements of $(C_6H_{11}NH_3)_2SbI_5$ and $(C_6H_{11}NH_3)_3[Sb_2I_9]\cdot I_2$.

Chemical formula	$C_{12}H_{28}I_5N_2Sb$	$C_{18}H_{28}I_{11}N_3Sb_2$
F.W.	956.61	2164.48
S.G.	*P 21/n*	*C 2/c*
a (Å)	13.8562(3)	8.5419(2)
b (Å)	8.7222(2)	21.9810(6)
c (Å)	20.5905(4)	23.3180(7)
α (deg)	90	90
β (deg)	95.2580(10)	90.193(1)
γ (deg)	90	90
V ($Å^3$)	2478.03(9)	4375 (2)
Z	4	4
D_c (g cm^{-3})	2.564	2.924
$F(000)$	1720.0	3368.0
Reflections coll./unique	29035/6130	81513/10410
R_{int}	0.0272	0.0627
GOF on F^2	1.085	1.041
R_1^a $[I > 2\sigma(I)]$	0.0483	0.0375
wR_2^b (all data)	0.1148	0.1650

^{a} $R_1 = \Sigma||F_o| - |F_c||/\Sigma|F_o|$; ^{b} $wR_2 = \Sigma[w(F_o^2 - F_c^2)^2]/\Sigma[w(F_o^2)^2]^{1/2}$

Table S2. Selected bond lengths (Å) and bond angles (°) for $(C_6H_{11}NH_3)_2SbI_5$.

Bond	Dist.
I(1)-Sb(1)	3.0724(7)
I(2)-Sb(1)	2.8497(7)
I(3)-Sb(1)	2.8093(7)
I(4)-Sb(1)	2.9774(7)
**Angle**	**(°)**
I(3)-Sb(1)-I(2)	97.77(3)
I(3)-Sb(1)-I(4)	90.58(2)
I(2)-Sb(1)-I(4)	95.03(2)
I(3)-Sb(1)-I(1)	94.11(2)
I(2)-Sb(1)-I(1)	91.70(2)
I(4)-Sb(1)-I(1)	171.22(2)

Symmetry transformations used to generate equivalent atoms: #1 x, -y+1/2, z #2 x, -y+3/2, z

Table S3. Selected bond lengths (Å) and bond angles (°) for $(C_6H_{11}NH_3)_3[Sb_2I_9]\cdot I_2$.

Bond	Dist.
I(1)-Sb(1)#2	3.1895(6)
I(1)-Sb(1)	3.1895(6)
I(2)-Sb(1)	2.9682(6)
I(3)-Sb(1)	2.8065(6)
I(4)-Sb(1)	2.8763(7)
I(5)-Sb(1)	3.0914(6)

Angle	(°)
I(3)-Sb(1)-I(4)	91.43(2)
I(3)-Sb(1)-I(2)	93.17(2)
I(4)-Sb(1)-I(2)	93.92(2)
I(3)-Sb(1)-I(5)	94.043(19)
I(4)-Sb(1)-I(5)	87.722(19)
I(2)-Sb(1)-I(5)	172.56(2)
I(3)-Sb(1)-I(1)	89.578(19)
I(4)-Sb(1)-I(1)	178.51(2)
I(2)-Sb(1)-I(1)	84.928(15)
I(5)-Sb(1)-I(1)	93.298(16)

Symmetry transformations used to generate equivalent atoms: #1 x, -y+1/2, z #2 x, -y+3/2, z

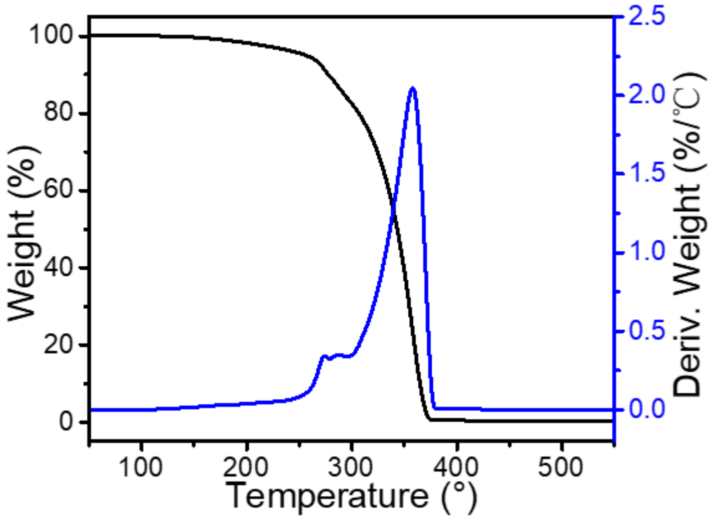

Figure S1.TGA curve (black) and DSC curve (blue) of $(C_6H_{11}NH_3)_3[Sb_2I_9]\cdot I_2$ powder sample.

Absorption spectrum

Ultraviolet-visible (UV–vis) diffuse reflectance spectroscopy measurements were

measured at room temperature from 200 to 1200 nm using a Shimadzu U-4100 spectrophotometer, with $BaSO_4$ powder utilized as the 100% reflectance standard. To determine the optical bandgaps, the UV-vis diffuse reflectance data were converted to absorbance using the Kubelka-Munk function: $F(R_\infty) = (1-R_\infty)^2/2\,R_\infty$. The bandgap energies (E_g) were then evaluated using the *Tauc* equation:

$$(h\nu \cdot F(R_\infty))^{1/n} = A(h\nu - E_g)$$

where h is Planck's constant, ν is the photon frequency, A is a proportionality constant, and E_g is the optical bandgap. The exponent n depends on the transition type: n = 1/2 for a direct transition and n = 2 for an indirect transition. The E_g values were obtained by plotting $(h\nu \cdot F(R_\infty))^{1/n}$ versus $h\nu$ and extrapolating the linear region to the energy axis.

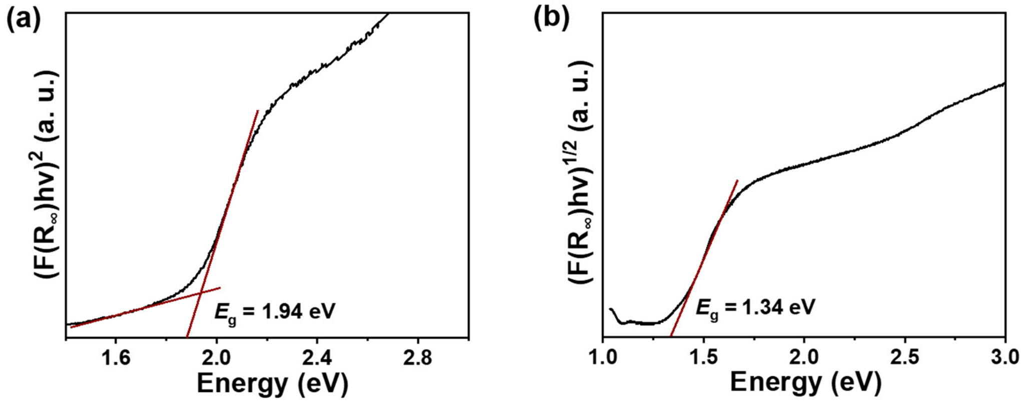

Figure S2. Tauc plots for the bandgap (E_g) of (a) $(C_6H_{11}NH_3)_2SbI_5$ and (b) $(C_6H_{11}NH_3)_3[Sb_2I_9]\cdot I_2$.

Raman spectroscopy

Raman measurements were performed in backscattering mode at room-temperature using a Horiba LabRAM HR Evolution spectrometer. A 633 nm He-Ne laser, focused to a ~2 μm spot via a 50 × Olympus objective (LMPlan FL, NA = 0.50), was used for excitation. To prevent sample degradation, the laser power kept at ~0.05 mW, with a spectral resolution of 0.1 μm^{-1}.

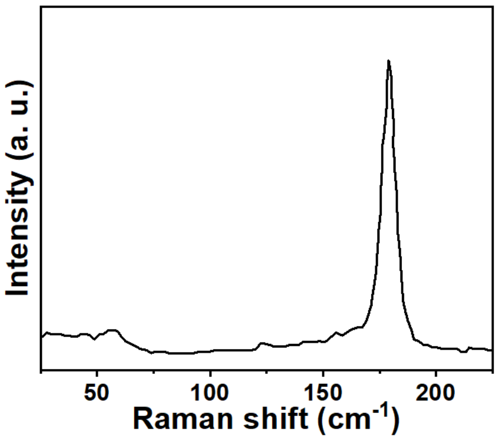

Figure S3. Raman spectrum of $(C_6H_{11}NH_3)_3[Sb_2I_9]\cdot I_2$.
